# Data on the clinical usefulness of brachial-ankle pulse wave velocity in patients with suspected coronary artery disease

**DOI:** 10.1016/j.dib.2017.12.028

**Published:** 2017-12-21

**Authors:** In-Chang Hwang, Kwang Nam Jin, Hack-Lyoung Kim, You-Nui Kim, Moon-Sun Im, Woo-Hyun Lim, Jae-Bin Seo, Sang-Hyun Kim, Joo-Hee Zo, Myung-A Kim

**Affiliations:** a Division of Cardiology, Department of Internal Medicine, Boramae Medical Center, Seoul National University College of Medicine, Seoul, Republic of Korea; b Department of Radiology, Boramae Medical Center, Seoul National University College of Medicine, Seoul, Republic of Korea

## Abstract

Brachial-artery pulse wave velocity (baPWV) is a simple and reliable tool for measurement of arterial stiffness. Our previous studies suggested that baPWV is associated with the presence and severity of coronary artery disease (CAD) and the risk of cardiovascular events. In the present data article, we provided supplementary data supporting the independent prognostic value of arterial stiffness, assessed by baPWV, in patients with suspected CAD (Hwang et al., 2017) [1]. The data was obtained from 523 patients undergoing coronary CT angiography (CCTA), and baPWV was measured at the time of CCTA. Patients with vulnerable coronary plaque or obstructive CAD on CCTA had higher age, more cardiovascular risk factors, and higher baPWV values. Given the significant association between high baPWV and the presence of vulnerable plaque or obstructive CAD as shown in this data article, the prognostic value of baPWV was further assessed in subgroups divided according to the CCTA findings (vulnerable plaque or obstructive CAD). In each subgroup by CCTA findings, multivariable Cox proportional hazard model analysis showed that high baPWV was an independent risk factor for cardiovascular events even after adjusting for clinical risk factors.

**Specifications Table**

**Table:** 

Subject area	*Cardiology*
More specific subject area	*Atherosclerosis and coronary artery disease*
Type of data	*Figures, tables and text*
How data was acquired	*Brachial-ankle pulse wave velocity (baPWV), coronary CT angiography (CCTA)*
Data format	*Analyzed*
Experimental factors	*Arterial stiffness was assessed by baPWV measurement, and coronary atherosclerosis was assessed by CCTA.*
Experimental features	*Occurrence of major adverse cardiovascular events was compared between subgroups divided according to the findings of baPWV and CCTA.*
Data source location	*Seoul, Republic of Korea*
Data accessibility	*The data is with this article*

**Value of the data**

• The data presented in this article provides evidence supporting the independent prognostic value of arterial stiffness to CCTA findings for the occurrence of cardiovascular events [1].
• The data also showed an excellent intra-observer correlation coefficient of 0.949 for baPWV [2].
• Together with our previous studies, these data supports clinical relevance of the arterial stiffness measured by baPWV among the patients with suspected CAD.

## 1 Data

The data for this paper was obtained from Boramae Medical Center, Seoul, Korea. In this hospital, the brachial-ankle pulse wave velocity (baPWV) is measured as part of the work-up protocol for patients at risk of cardiovascular disease, based on the evidence supporting the clinical usefulness of baPWV. According to our previous studies, arterial stiffness measured by baPWV is correlated with left ventricular (LV) diastolic function [3], [4], [5], and LV global longitudinal strain [6]. Also, we demonstrated that the increased arterial stiffness is associated with the presence and severity of coronary artery disease (CAD) [7], [8].

Adding to the previous studies, we investigated the prognostic value of baPWV in patients with suspected CAD and found that the increased arterial stiffness has an independent prognostic value even after adjusting for clinical risk factors and CCTA findings. These results were recently published in *Atherosclerosis* as a research article titled “*Additional Prognostic Value of Brachial-Ankle Pulse Wave Velocity to Coronary Computed Tomography Angiography in Patients with Suspected Coronary Artery Disease*”, and in this data article, we present the supplementary results supporting the original article [1]. Supplementary Fig. S1 shows adjusted event-free survival curves of subgroups divided according to the differential cutoff values of baPWV and CCTA findings. Cutoff values of baPWV obtained from receiver-operating characteristic (ROC) curve analyses were 1612.5 cm/s for the patients with <50% stenosis, 1630.0 cm/s for those with ≥50% stenosis, 1591.5 cm/s for those without vulnerable plaque, and 1630.0 cm/s for those with vulnerable plaque on CCTA. The prognostic significance of high baPWV for cardiovascular events remained unchanged when the cutoff values of baPWV were differentially applied in these subgroups. In Supplementary Table S1, baseline characteristics of study population are shown according to the presence of vulnerable plaque (partially-calcified or non-calcified plaques) on CCTA (left columns) and the presence of obstructive CAD (≥50% stenosis) on CCTA (right columns). Supplementary Table S2 presents the univariable predictors for the presence of vulnerable coronary plaque or obstructive CAD. The univariable factors with *P* values <0.100 entered the multivariable logistic regression analyses with stepwise backward elimination methods, as shown in our recent study [1].

## 2 Reproducibility studies

The clinical usefulness of baPWV is not only based on its independent prognostic value for the risk of cardiovascular events but also related to the simplicity and reliability of the measurement [9]. In our institute, baPWV measurements are being performed by a single experienced operator. The reliability of baPWV measurement was analyzed from randomly selected 50 patients, using intra-observer correlation coefficient (ICC) and Bland–Altman statistics (Supplementary Fig. S2). The ICC of baPWV measurement was 0.949 (95% confidence interval, 0.911–0.971; *P*<0.001), and the bias for intra-observer measurement was 3.72 cm/s (95% levels of agreement, −136.1 to 143.6 cm/s) [2].
