# Supplementary material for: Data on the clinical usefulness of brachial-ankle pulse wave velocity in patients with suspected coronary artery disease
Source: Data Brief. 2017 Dec 21;16:1078–82. doi: 10.1016/j.dib.2017.12.028 (PMC5767841; doi:10.1016/j.dib.2017.12.028)
Supplement: Supplementary file 2 — Supplementary material [file mmc2.pdf]

Supplementary Figure S1

A

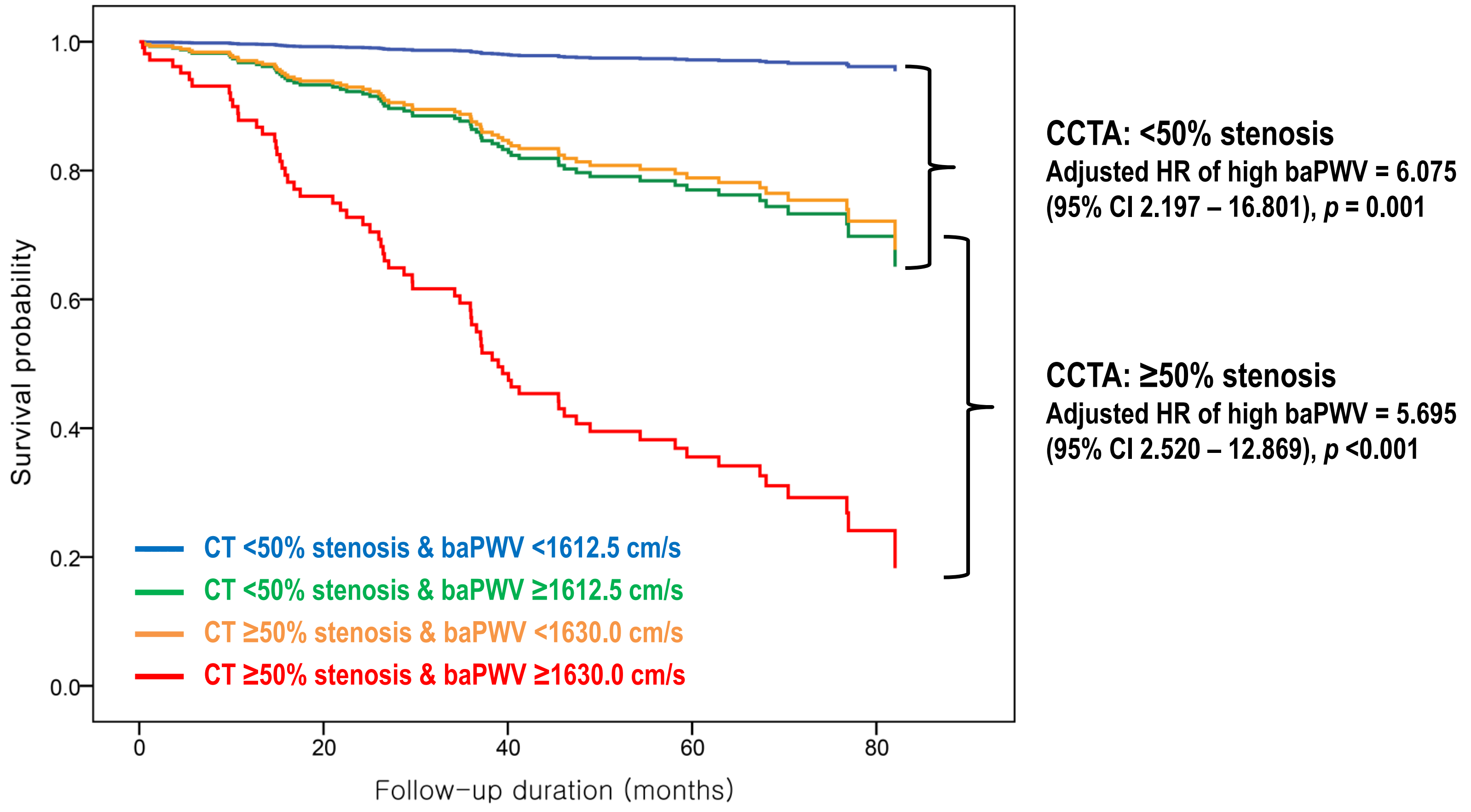

B

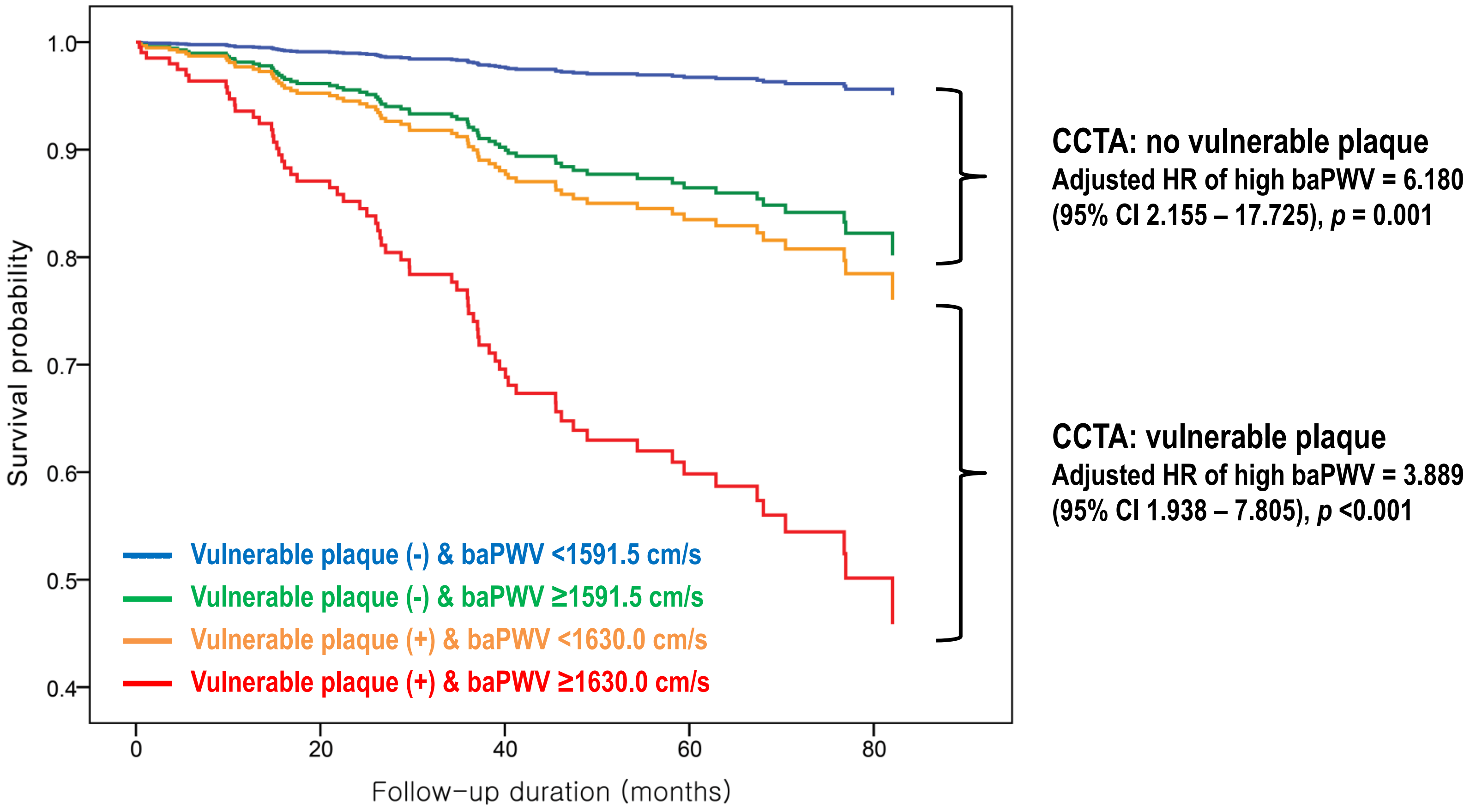

Supplementary Figure S1. Risk-adjusted event-free survival according to the differential cutoff values of baPWV and CCTA findings
(A) Adjusted event-free survival curves for the composite endpoint according to the differential baPWV cutoff values (1612.5 cm/sec for patients with <50% stenosis, and 1630.0 cm/sec for patients with ≥50% stenosis) and the degree of coronary artery stenosis on CCTA (≥50% vs. <50% stenosis). **(B)** Adjusted event-free survival curves according to the differential baPWV cutoff values (1591.5 cm/sec for patients without vulnerable plaque, and 1630.0 cm/sec for patients with vulnerable plaque) and the presence of vulnerable plaques on CCTA (PCP or NCP). Survival curves were adjusted for significant univariable predictors, such as age, sex, hypertension, DM, dyslipidemia, CKD, HF, AF, use of ACEi, and anemia. Abbreviations: baPWV, brachial-ankle pulse wave velocity; CCTA, coronary CT angiography; PCP, partially calcified plaque; NCP, noncalcified plaque; SSS, stenosis severity index; HR, hazard ratio; CI, confidence interval.
