# Supplementary material for: Data on the clinical usefulness of brachial-ankle pulse wave velocity in patients with suspected coronary artery disease
Source: Data Brief. 2017 Dec 21;16:1078–82. doi: 10.1016/j.dib.2017.12.028 (PMC5767841; doi:10.1016/j.dib.2017.12.028)
Supplement: Supplementary file 3 — Supplementary material [file mmc3.pdf]

Supplementary Figure S2

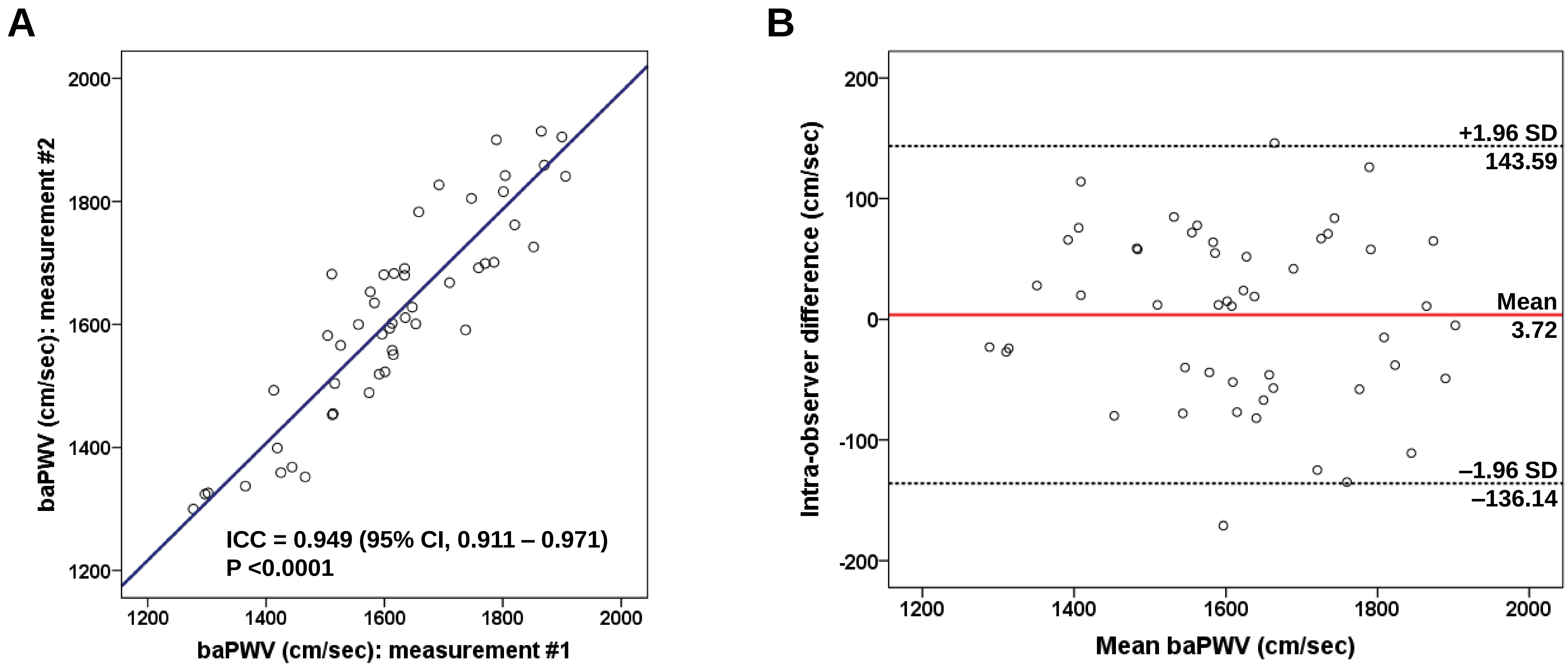

Supplementary Figure S2. Intra-observer variability of baPWV measurement

(A) Correlations displaying the intra-observer variability. **(B)** Bland-Altman graph for baPWV measurement.

Abbreviations: baPWV, brachial-ankle pulse wave velocity; ICC, intra-observer correlation coefficient; SD, standard deviation.
